# Performance study of the anterior nasal AMP SARS-CoV-2 rapid antigen test in comparison with nasopharyngeal rRT-PCR

**DOI:** 10.1099/acmi.0.000361

**Published:** 2022-06-01

**Authors:** Georg Leixner, Astrid Voill-Glaninger, Isabella Krejci, Julia Gaugeler-Kurzweil, Tanja Kusstatscher, Walter Krugluger, André Viveiros

**Affiliations:** ^1^​ Institute for Laboratory Medicine, Klinik Landstrasse, Vienna Healthcare Group, Juchgasse 25, 1030, Vienna, Austria; ^2^​ Institute for Laboratory Medicine, Klinik Donaustadt, Vienna Healthcare Group, Langobardenstrasse 122, 1220 Vienna, Austria

**Keywords:** RT-PCR, COVID-19, anterior nasal testing, viral load, antigen expression, coronavirus

## Abstract

**Introduction.:**

The gold standard for severe acute respiratory syndrome coronavirus 2 (SARS-CoV-2) detection is real-time reverse transcription PCR (rRT-PCR), which is expensive, has a long turnaround time and requires special equipment and trained personnel. Nasopharyngeal swabs are uncomfortable, not suitable for certain patient groups and do not allow self-testing. Convenient, well-tolerated rapid antigen tests (RATs) for SARS-CoV-2 detection are called for.

**Gap statement.:**

More real-life performance data on anterior nasal RATs are required.

**Aim.:**

We set out to evaluate the anterior nasal AMP RAT in comparison with rRT-PCR in a hospital cohort.

**Methodology.:**

The study included 175 patients, either hospitalized in a coronavirus disease 2019 (COVID-19) ward or screened in a preadmittance outpatient clinic. Two swabs were collected per patient: an anterior nasal one for the RAT and a combined naso-/oropharyngeal one for the rRT-PCR. Sixty-five patients (37%) were rRT-PCR-positive [cycle threshold (*C*_t_) <40].

**Results.:**

The anterior nasal AMP RAT showed an overall sensitivity and specificity of 29.2 % (18.6–41.8, 95 % CI) and 100.0 % (96.7–100.0, 95 % CI) respectively. In patients with a *C*_t_ value <25, <30 and <33, higher sensitivities were observed. Time since symptom onset was significantly higher in patients with a false-negative RAT (*P*=0.02).

**Conclusion.:**

The anterior nasal AMP RAT showed low sensitivities in this cohort, especially in patients with a longer time since symptom onset. Further knowledge concerning the viral load and antigen expression over time and in different swabbing locations is needed to outline the usage time frame for SARS-CoV-2 RAT.

## Introduction

Nucleic acid amplification tests (NAATs) such as real-time reverse transcription PCR (rRT-PCR) are the gold standard for the diagnosis of severe acute respiratory syndrome coronavirus 2 (SARS-CoV-2) [1, 2]. In ambulatory patients, naso- and oropharyngeal swabs (or washes) are recommended as testing specimens [3]. Challenging aspects of rRT-PCR testing include specimen transport and storage, as well as being a cost- and labour-intensive technology that requires trained personnel [4, 5]. Point-of-care testing (POCT) rRT-PCR platforms enable easier and faster diagnostics. However, their use in low-income countries and during disease outbreaks is limited by their high cost and long turnaround times [6]. Rapid antigen tests (RATs) represent a practical and cheap alternative for swift isolation of highly infectious cases, enabling the reduction of further transmission and quick contact tracing [7, 8]. The fact that no refrigeration is needed for storage and that RATs have a long shelf life makes its use ideal in small ambulatory care settings, developing countries, remote regions and battlefields [9]. Although vaccination programmes have been established, the diagnosis and management of coronavirus disease 2019 (COVID-19) remains a highly relevant issue [10]. Independently of the future of the pandemic, SARS-CoV-2 testing will probably be a mainstay of the differential diagnosis of respiratory tract infections.

When NAATs are not available or feasible, SARS-CoV-2 RAT should guarantee a minimum performance of ≥80 % sensitivity and ≥97 % specificity [11]. There are a substantial number of commercially available RATs [12], some of which have been reviewed recently by Cochrane [13]. However, only 28 have approval from the US Food and Drug Administration (FDA) as of 23 June 2021 [14].

Nasopharyngeal swabbing is associated with discomfort, is not feasible in certain patient groups (e.g. children) and requires a trained healthcare provider [4]. To overcome these limitations and allow self-testing, manufacturers have developed minimally invasive RATs through nasal mid-turbinate or anterior nasal swabbing [12]. Nasal mid-turbinate swabs are inserted in a parallel position to the palate until resistance is met at the turbinates, preferably with the patient’s head tilted 70°, whereas anterior nasal specimens are collected by inserting the swab upwards, with no head tilting being necessary [4].

We recently evaluated the nasopharyngeal AMP Rapid Test SARS-CoV-2 Ag, where the sensitivity varied *C*_t_-dependently from 69.2–100 % [15]. Here, we aimed to study the performance of the commercially available anterior nasal AMP Rapid Test SARS-CoV-2 Ag in a hospital cohort [12, 16].

## Methods

### Patients

**Fig. 1.**
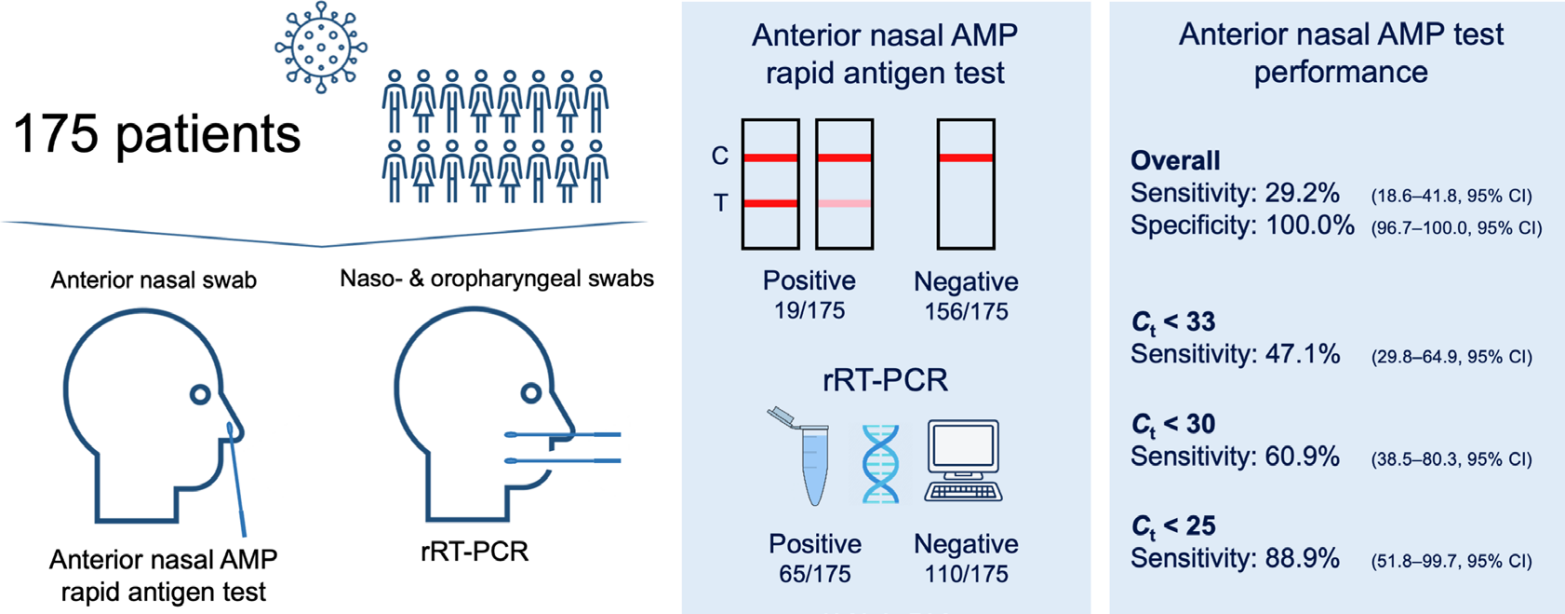
Study design and anterior nasal AMP RAT performance data.

A total of 175 adult patients (≥18 years old) who presented at the Klinik Landstrasse, a tertiary hospital in Vienna, Austria, from 9 April 2021 through 25 May 2021, were included in this retrospective study (Fig. 1). The study cohort was assembled from 2 different patient groups: (i) 78 inpatients from a COVID-19 ward and (ii) 97 asymptomatic patients who were routinely swabbed at a COVID-19 preadmittance screening outpatient department. In both cases, an anterior nasal RAT had been carried out prior to the rRT-PCR to shorten the time to diagnosis during the third high-COVID-19-incidence phase (‘third wave’) in Austria. The COVID-19 ward patients had been diagnosed through a positive rRT-PCR, performed in either an external laboratory or in a POCT setting at our emergency unit. According to the hospital’s infectious disease policy, all patients, regardless of the ward and symptoms, had to be rRT-PCR tested for SARS-CoV-2 once every 5 days of hospitalization. For patient discharge, a rRT-PCR with a cycle threshold (*C*_t_) value >30, along with clinical criteria, had to be achieved.

Anterior nasal swabs for RATs had been collected immediately before the combined naso- and oropharyngeal swabs for rRT-PCR. Every healthcare provider was instructed for both anterior nasal and naso- oropharyngeal sampling and so the bias inherent to all swabbing techniques was minimized. While the RAT had been performed right away by a healthcare provider and read after 15 min, the rRT-PCR was carried out subsequently and within a period of 4 h in our laboratory. Therefore, rRT-PCR results were unknown at the time of RAT performance and reading.

Patient records were retrospectively analysed for clinical and demographic data collection. COVID-19 symptoms were classified according to the World Health Organization (WHO) classification [17] into (i) most common (fever, dry cough, fatigue), (ii) less common (sore throat, diarrhoea, headache or other aches/pains, conjunctivitis, anosmia or ageusia, skin rash, discolouration of fingers or toes) and (iii) serious (dyspnoea, chest pain, focal neurological deficit).

### AMP rapid test SARS-CoV-2 Ag for anterior nasal swabs

The AMP Rapid Test SARS-CoV-2 Ag for anterior nasal specimens (AMP Diagnostics, AMEDA Labordiagnostik GmbH, Graz, Austria), henceforth anterior nasal AMP RAT, is an EU CE/IVD-certified lateral flow immunochromatographic test for qualitative detection of SARS-CoV-2. Because the anterior nasal AMP RAT detects the nucleocapsid protein, spike protein variants have no influence on the test [16]. Specimen collection and testing were carried out according to the manufacturer’s instructions. For anterior nasal sampling, the swab was inserted upwards approximately 2 cm in each nostril and rotated 5–10 times against the nasal wall. Afterwards, the swab was inserted, tip first, in the extraction buffer tube and rotated a minimum of six times while gently pressing the head of the swab against the inner wall of the buffer tube. After 1 min of incubation, four drops of the extraction solution were given into the sample well of the cassette, which then migrated by capillary effect along the membrane. In the presence of the nucleocapsid antigen, monoclonal antibodies conjugated with colloidal gold particles were then captured by secondary monoclonal antibodies and immobilized in the test region. An internal control (IC) line must appear for the test to be valid. Test results can be read after 15 min but not longer than 20 min. Specimen collection and analysis were performed at room temperature in a point-of-care setting.

### rRT-PCR

Combined naso- and oropharyngeal swabs with a flocked head were collected for the rRT-PCR and inoculated in a sterile 2 ml 0.85 % NaCl solution for the Kirby–Bauer method (Axonlab AG, Stuttgart, Germany). All samples were analysed within a period of 4 h. The RNA extraction was carried out with the MagNA Pure 24 (Roche Diagnostics, Rotkreuz, Switzerland) and the rRT-PCR was performed on the cobas z480 (Roche Diagnostics, Rotkreuz, Switzerland) with the ViroReal Kit SARS-CoV-2 Multiplex (Ingenetix GmbH, Vienna, Austria). The latter targets the N and the RdRp genes. All rRT-PCR results with a *C*_t_ value <40 were considered positive and a mean *C*_t_ value was calculated. As rRT-PCR is the diagnostic gold standard for SARS-CoV-2 detection, positive and negative samples were considered as true positive and true negative. Here, we evaluated the clinical sensitivity and specificity, which included the material examined and the timing of sampling.

For epidemiological and surveillance reasons, 64 of 65 positive rRT-PCR samples were genotyped in the laboratory of the Klinik Donaustadt, a partnering laboratory within the Vienna Healthcare Group. The RNA extraction was performed with a QIAsymphony SP (Qiagen, Hilden, Germany) and genotyping was carried out using the assay kits VirSNip SARS del69,70+484K+501Y and VirSNip SARS Spike 417T 681 h (TIB MOLBIOL Syntheselabor GMBH, Berlin, Germany). The rRT-PCR was conducted with the Rotor-Gene Q (Qiagen, Hilden, Germany).

### Statistics

IBM SPSS Statistics version 22.0.0.1 (IBM Corp., Armonk, NY, USA) and MedCalc version 17.7.2. (MedCalc Software, Ostend, Belgium) were used for statistical analysis. A significance value of 0.05 was considered in all statistical tests. The Kolmogorov–Smirnov test was calculated for normality of distribution and variables that were not normally distributed were reanalysed after logarithmic transformation. Continuous variables were reported using median, 25th and 75th percentiles and analysed using Student’s *t*-test or the Mann–Whitney U test, as appropriate. Categorical variables were expressed in frequencies (with percentages in parenthesis) and tested for significance using the χ² test or Fisher’s extract test. A univariate and multivariate binary logistic regression model was used for the prediction of RAT positivity (dependent or outcome variable). *C*_t_ value and time since symptom onset were used as independent variables, as these have been shown to be associated with positive RATs in other studies [13].

We grouped patients according to three *C*_t_ cut-offs: 25, 30 and 33. Thirty is the most relevant cut-off in Austria, as it is used for epidemiological and clinical decision-making. The Austrian Federal Ministry of Social Affairs, Health, Care and Consumer Protection published a recommendation for discharging COVID-19 patients out of isolation according to clinical criteria and if follow-up *C*_t_ values are >30 [18, 19]. Further, a recent Cochrane review of SARS-CoV-2 POCT used the cut-offs 25 and 33 for RAT classification [13].

## Results

In this study, we included 175 symptomatic and asymptomatic patients and compared the anterior nasal AMP RAT results with the rRT-PCR. Seventy-eight inpatients were hospitalized in a COVID-19 ward and 97 were screened in a COVID-19 preadmittance outpatient clinic. The median patient age of the entire cohort was 65 and no sex differences were noted. Twenty-seven per cent (47/175) of all patients were symptomatic, 22 % (39/175) presented with severe symptoms and 23 % (40/175) needed oxygen support (Table 1). Patients who were screened at the preadmittance outpatient clinic were asymptomatic and significantly younger than the COVID-19 inpatients (Table S1, available in the online version of this article).

**Table 1.** Demographic and clinical data for patients

	All patients *n*=175	rRT-PCR-positive *n*=65	rRT-PCR-negative *n*=110	*P* value	RAT-positive *n*=19	RAT-negative *n*=46	*P* value
Age, years	65 (46–75)	72 (60–76)	59 (42–69)	<0.001	75 (62–77)	72 (60–75)	0.39
Sex, female/male (%)	99/76 (57/43)	35/30 (54/46)	64/46 (58/42)	0.58	13/6 (68/32)	22/24 (48/52)	0.13
Symptoms*, *n* (%)							
None Most common Less common Severe	128 (73) 21 (12) 3 (2) 39 (22)	28 (43) 18 (28) 2 (3) 30 (46)	100 (91) 3 (3) 1 (1) 9 (8)	<0.001	6 (32) 9 (47) 0 (0) 10 (53)	22 (48) 9 (20) 2 (4) 20 (43)	0.34
Oxygen therapy, *n* (%)	40 (23)	32 (49)	8 (7)	<0.001	11 (58)	21 (46)	0.37
Time since symptom onset, days	14 (7–18)	12 (7–17)	14 (11–20)	0.22	9 (5–12)	15 (7–18)	0.02

Data are given as *n* (%) or median (25th–75th percentiles)

*Symptoms were grouped according to the WHO classification [17] into most common (fever, dry cough, fatigue), less common (sore throat, diarrhoea, headache or other aches/pains, conjunctivitis, anosmia or ageusia, skin rash, discolouration of fingers or toes) and severe (dyspnoea, chest pain, focal neurological deficit). These were counted separately, as a combination of symptoms from different groups is possible.

The rRT-PCR results revealed 65 positive samples and 110 negative samples. The median *C*_t_ value was 32.2 (range: 16.1–39.9). As shown in Table 1, median time since symptom onset to testing was not statistically different between rRT-PCR-positive and -negative patients (12 and 14 days, respectively).

The alpha variant (B1.1.7) was detected in all genotyped rRT-PCR-positive samples and, therefore, false-positive rRT-PCR results could be excluded.

**Fig. 2.**
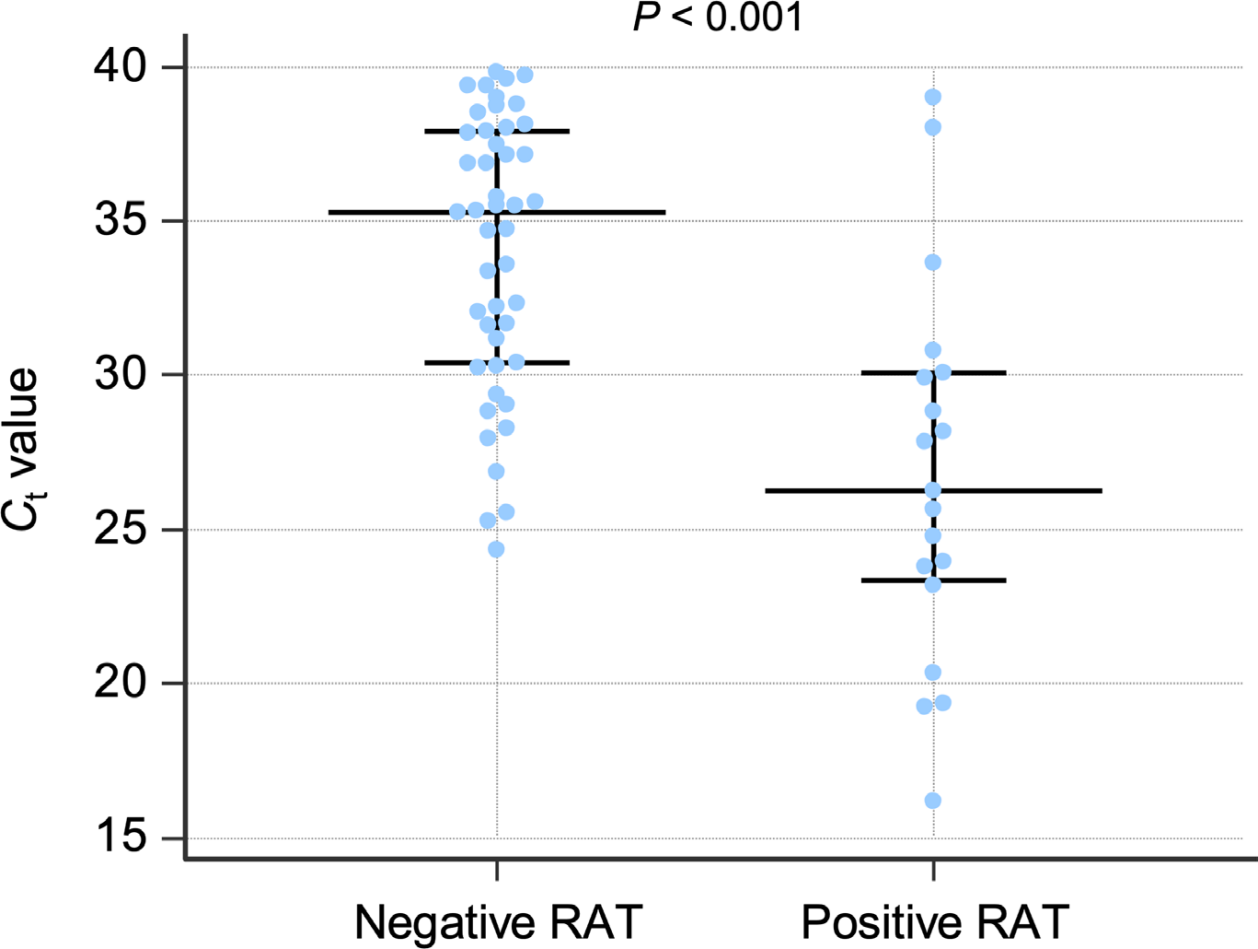
*C*_t_ values (abscissa) according to the anterior nasal AMP RAT (ordinate). Each blue dot represents a single rRT-PCR test. Median, 25th and 75th percentiles are shown in black. The median *C*_t_ value was 35.3 (30.4–37.9) in the negative RAT group and 26.2 (23.3–30.0) in the positive RAT group.

In all anterior nasal AMP RATs performed, the IC was positive and so no test had to be repeated. The anterior nasal AMP RAT showed an overall sensitivity of 29.2 % (18.6–41.8, 95 % CI) and a specificity of 100.0 % (96.7–100.0, 95 % CI). rRT-PCR-positive patients were grouped according to a *C*_t_ value <33, <30 and <25, in which case the anterior nasal AMP RAT sensitivity improved to 47.1 % (29.8–64.9, 95 % CI), 60.9 % (38.5–80.3 %, 95 % CI) and 88.9 % (51.8–99.7, 95 % CI) (Fig. 1). The median *C*_t_ value was 35 in patients with a negative RAT and 26 in patients with a positive RAT (Fig. 2).

When comparing RAT-positive and -negative patients, no differences were found in age, sex, symptoms or need for oxygen therapy. Time since symptom onset was significantly higher in patients with a negative RAT (Table 1). However, in a binary logistic regression model in the rRT-PCR-positive group, we could identify the *C*_t_ value, but not time since symptom onset, as an independent predictor for a positive anterior nasal AMP RAT (Table 2).

**Table 2.** Binary logistic regression to estimate RAT positivity in rRT-PCR-positive patients

	rRT-PCR positive *n*=65			
	Univariate OR (95 % CI)	*P* value	Multivariate OR (95 % CI)	*P* value
*C*_t_ value	0.769 (0.670–0.882)	<0.001	0.756 (0.653–0.874)	<0.001
Time since symptom onset	0.995 (0.961–1.030)	0.77	1.019 (0.982–1.057)	0.32

## Discussion

In the instructions for the AMP RAT, sensitivity and specificity are only given for nasopharyngeal samples (97.3 and 100 %, respectively). An additional statement discloses a decrease of approximately 5 % when using anterior nasal swabs [16]. Here, the anterior nasal AMP RAT showed a low overall sensitivity of 29.2 %. We recently evaluated the test performance of the nasopharyngeal kit of the AMP RAT in a hospital setting and reported an overall sensitivity of 69.2 %, again lower than the one disclosed in the manufacturer’s instructions [15]. The test cassette and buffer are identical in the anterior nasal and nasopharyngeal kits. Therefore, the dissimilarity between the real-life performance of the nasopharyngeal and the anterior nasal AMP RAT could be explained by sample acquisition itself. Similarly, another real-life study has described a lower level of performance for anterior nasal testing when compared to nasopharyngeal sampling [20].

Other published data on nasal RATs have disclosed higher sensitivities but have predominantly included patients with low *C*_t_ values [21–24]. In our study on anterior nasal samples, the median *C*_t_ value was 32.2 and 65 % of rRT-PCR positive patients (42/65) had a *C*_t_ value ≥30 (Fig. 1, Table 1). Further, 43 % (28/65) of all rRT-PCR-positive patients were asymptomatic. Both factors could justify the low overall sensitivity presented here.

The gold standard of SARS-CoV-2 detection remains the rRT-PCR. However, the use of RATs is particularly important whenever POCT rRT-PCR platforms are not available (e.g. small ambulatory care settings, developing countries). In this case, and according to the WHO, RATs should have a sensitivity ≥80 % and a specificity ≥97 % [11]. In conformity with previous studies on nasopharyngeal and anterior nasal RATs, the sensitivity of the anterior nasal AMP increased with lower *C*_t_ values (Fig. 1) [15, 21–28] and a sensitivity ≥80 % could be found in patients with a *C*_t_ value <25.

Data from 133 RAT studies were recently reported in a meta-analysis and showed that anterior nasal and nasopharyngeal RATs have comparable sensitivities (75.5%, 95 % CI: 70.4–79.9 % for anterior nasal and 71.6 %, 95 % CI: 68.1–74.9 % for nasopharyngeal RATs) [29]. For this reason, and considering that nasopharyngeal swabs cause more discomfort and present an increased risk of complications compared to other types of nasal swabs, anterior nasal swabbing might become the mainstay of SARS-CoV-2 antigen testing [30–32]. Furthermore, anterior nasal sampling allows self-testing, since no particular understanding of nasal anatomy is needed.

Robust data have shown that RAT sensitivity is highest in the first week after symptom onset and in patients with *C*_t_≤25 [13, 29]. In accordance, our study showed a decreasing clinical sensitivity over time as time since symptom onset was significantly higher in patients with a false-negative RAT. We therefore recommend performing anterior nasal RATs in early symptomatic patients in order to improve RAT sensitivity. This study sheds some light on RAT limitations and might help to narrow down the utility of antigen testing. Studies on the viral load and antigen expression in different locations of the upper respiratory tract over time are needed. Such a head-to-head comparison will allow us to deconvolute the contribution of sampling to the clinical sensitivity of SARS-CoV-2 RATs and define the exact time frame where the use of such RATs is indicated.

## Supplementary Data

Supplementary material 1
