# Supplementary material for: Performance study of the anterior nasal AMP SARS-CoV-2 rapid antigen test in comparison with nasopharyngeal rRT-PCR
Source: Access Microbiol. 2022 Jun 1;4(6):acmi000361. doi: 10.1099/acmi.0.000361 (PMC9394671; doi:10.1099/acmi.0.000361)
Supplement: Supplementary material 1 [file acmi-4-361-s001.pdf]

Supplement

Table S1. Demographic and clinical data of patients.

	**All patients n = 175**	**COVID-19 ward n = 78**	**Pre-admittance outpatient clinic n = 97**	***P* value**
Age, years	65 (46 – 75)	72 (53 – 76)	58 (42 – 68)	0.001
Sex, female/male (%)	99/76 (57/43)	40/38 (51/49)	59/38 (61/39)	0.21
Symptoms*, n (%)				< 0.001
None	128 (73)	31 (40)	0	
Most common	21 (12)	21 (27)		
Less common	3 (2)	3 (4)		
Severe	39 (22)	39 (50)		
Oxygen therapy, n (%)	40 (23)	40 (45)	0	< 0.001
Time since symptom onset, days	14 (7 – 18)	14 (7 – 18)	n.a.	n.a.

Data are given as n (%) or median (25th – 75th percentiles)

* Symptoms were grouped according to the WHO classification (1) in common (fever, dry cough, fatigue), less common (sore throat, diarrhea, headache or other aches/pains, conjunctivitis, anosmia or ageusia, skin rash, discoloration of fingers or toes) and severe (dyspnea, chest pain, focal neurological deficit). These were counted separately, as a combination of symptoms from different groups is possible.

n.a.: non-applicable as all patients screened at the pre-admittance outpatient clinic were asymptomatic

Reference

1. WHO. Coronavirus, Symptoms 2021 [Available from: https://www.who.int/health-topics/coronavirus#tab=tab_3.
